# An Ascorbate Bluetooth© Analyzer for Quality Control of Fresh-Cut Parsley Supply Chain

**DOI:** 10.3390/antiox10091485

**Published:** 2021-09-17

**Authors:** Ylenia Spissu, Antonio Barberis, Guy D’hallewin, Germano Orrù, Alessandra Scano, Gavina Rita Serra, Milo Pinna, Cristian Pinna, Salvatore Marceddu, Pier Andrea Serra

**Affiliations:** 1 Institute of Sciences of Food Production, National Research Council, 07100 Sassari, Italy; yspissu82@gmail.com (Y.S.); guy.dhallewin@cnr.it (G.D.); orru@unica.it (G.O.); gavina.serra@cnr.it (G.R.S.); salvatore.marceddu@cnr.it (S.M.); paserra@uniss.it (P.A.S.); 2 Department of Surgical Sciences, Molecular Biology Service, University of Cagliari, 09042 Cagliari, Italy; alessandrascano@libero.it; 3 La Linea Verde Soc. Agricola S.P.A., 09094 Brescia, Italy; milo.pinna@lalineaverde.it; 4 Fresco & Pronto S.r.l., 09042 Cagliari, Italy; frescoeprontosrl@gmail.com; 5 Department of Medical, Surgical and Experimental Medicine, Medical School, University of Sassari, 07100 Sassari, Italy

**Keywords:** Bluetooth©, ascorbic acid, screen-printed sensors, fresh-cut produce, parsley, ascorbate oxidase, ascorbate peroxidase

## Abstract

This work provides companies in the fresh-cut produce sector with an Ascorbate Bluetooth© Analyzer (ABA), a screen-printed sensor-based device for ascorbic acid (AA) detection, for quality control all along the supply chain. The amperometric detection of AA on fresh and fresh-cut parsley, under correct and incorrect storage temperature, allowed us to investigate the kinetics of AA decay in response to oxidative stress. The role of ascorbate oxidase (AOx) and ascorbate peroxidase (APx) was studied. ABA was used in situ by unskilled personnel. Treatments influenced AA decay kinetics, which were linear in fresh parsley, and non-linear in fresh-cut. Two hours at 28 °C immediately after chopping, the resilience of the fresh-cut parsley was reduced, even though the cold chain was restored. Two hours at −2 °C caused a rapid loss of AA until its complete decay after 72 h. Significant differences between treatments were observed in both the expression and activity of AOx and APx. ABA registered sudden changes of parsley AA following unpredicted variations of temperature during processing or transport. It was useful to remedy the effects of unexpected flaws in the cold chain, which can be proposed for quality preservation of different fresh-cut produce.

## 1. Introduction

Fresh-cut vegetables are widely consumed because they do not require preparation time, which make them perfectly compatible with the current timing of lunch breaks [1]. As is well known, however, they undergo rapid and inevitable deterioration, which reduces their use and marketability to only a few days and always requires that they are processed, transported and stored in full compliance with the cold chain [2,3]. It is equally clear that the cutting procedure during fresh-cut processing induces oxidative stress with accumulation of reactive oxygen species (ROS) that accelerate the processes of senescence [4]. Notwithstanding that it is not possible to stop the decay process [5], the control of temperature is crucial to delay the quality depletion and to guarantee the shelf life of fresh-cut vegetables [6]. Despite precise laws and regulations, unpredictable but undeniable flaws in the maintenance of the correct operating temperature exist at all levels of the supply chain, including processing, transport and storage. These flaws influence the quality of the produce without any immediate variation in the visual or sensory properties, and the quality indicators recognized by law, such as the microbiological charge [7,8], enzymatic browning or unpleasant odors [2,9], highlight the effects of damage with such a delay that no effective intervention is possible to remedy it. There are no immediate detection systems, based on quality indicators, of the damage caused by interruption of the cold chain.

The concentration of ascorbic acid (AA), an effective radical scavenger of ROS produced by oxidative stress, is recognized by the scientific community as a valid indicator of quality depletion, since it rapidly varies according to several post-harvest factors [10,11,12]. Its detection in fresh-cut produce is easy, and its high reducing power due to its low redox potential makes it a faster antioxidant than most of the other molecules in fruit and vegetables [13,14,15,16]. However, it is not always easy to understand when AA consumption, usually observed during the shelf-life of fresh-cut vegetables and fruit [17,18], is due to its ROS scavenging activity, or whether it is the main substrate of the activity of enzymes, such as ascorbate oxidase (AOx) and ascorbate peroxidase (APx), which are activated in response to wounding or incorrect storage temperatures. AOx has been found mainly in the apoplastic space of cells of several plants, most notably the Cucurbitaceae family. There, it converts AA to monodehydroascorbate by reducing oxygen to two water molecules, without producing ROS or H_2_O_2_ as side-products [19]. AOx and its different isoforms deserve more study, as their gene expression is induced or repressed as a result of different oxidative stresses, such as heat or wounding [20,21], or induced and repressed in response to the same stress [11]. APx is part of a pool of enzymes which, together with AA and glutathione, constitute the ascorbate–glutathione cycle, active in the cell organelles of various plants as a scavenger of H_2_O_2_. Previous publications indicated that cutting procedures can induce, as in tomatoes [22], or repress, as in sliced potatoes [23], the expression of genes that encode for the enzymes of the aforementioned cycle, with consequent accumulation or decrease of the AA level in the tissues of these species. Different results therefore suggest that the stress response is different in different plants and plant tissues, and that it depends on the intensity of cutting and on the mechanisms that can be put in place to control processing and storage temperatures.

Compared to titrimetric or chromatographic detection methods [24], the sensor-based real time electrochemical detection of AA has been demonstrated to be a reliable tool to evaluate the oxidative stability and the nutritional quality of fresh-cut fruit, as well as to investigate the activity of the enzymatic systems involved in AA metabolism [17]. Today, in spite of the fact that the fresh-cut industry is in urgent need of new and improved technologies for shelf-life extension [2], AA does not appear on the list of rules, guidelines or regulations requiring mandatory compliance.

On the other hand, telemetry allows processes to be monitored at a distance and in inaccessible or potentially dangerous environments. Our group has recently proposed several telemetry devices, integrated within electrochemical analyzers, for the wireless monitoring of parameters of particular interest both in terms of food quality [13,17] and during some production processes [25] or in vitro experimental conditions [26]. The logical consequence of these studies is the implementation of telemetry with the most used communication technologies like ZigBee©, Wi-Fi, radio-frequency identification (RFID) and Bluetooth© low energy (BLE). The implementation of IoT for food quality monitoring has exceptional potential, but the real applications of IoT for food quality monitoring are still rare [27]. Bluetooth© connection in particular is exceptionally effective for short range wireless communication and has already been proposed for controlling temperature and humidity in food storage systems [28].

In this work, a very simple and inexpensive Bluetooth©-based system, called Ascorbate Bluetooth© Analyzer (ABA), is proposed to acquire real-time information on the decay kinetics of AA in fresh-cut parsley. The screen-printed sensors (SPEs) were combined with ABA for this purpose, since they combine robustness with a very high repeatability of the measurements. As a matter of fact, the printing technology has appeared over the past twenty years as the most satisfactory methodology for mass production of reliable disposable analytical devices [29], and SPEs enable simple integration and the portability needed for on-field applications [30]. The role of enzymatic systems that use ascorbate as the substrate was also investigated to understand which systems are responsible for the progressive reduction of the AA concentration that inevitably accompanies the qualitative decay of this minimally processed species [31]. The effect of incorrect storage temperature in critical points of the fresh-cut parsley supply chain was emphasized, and the consumption of AA and the activity of AOx and APx were monitored from the moment of chopping throughout the shelf-life.

Fresh-cut parsley (*Petroselinum crispum* (Mill.) Fuss), a ready-to-use ingredient or garnish for multiple dishes in home kitchens and restaurants, deserves interest for the beneficial effects that its main compounds exert on health [32,33,34]. It was chosen for this study because it is particularly rich in AA [31], and, compared to other minimally processed produce, it undergoes more rapid deterioration because of chopping, a mechanical wounding that causes a tremendous disruption of cells, inducing a release of nutrients, accelerating senescence and quality loss [4,10], enhancing bacterial growth [35] and, on the whole, strongly affecting its shelf-life.

## 2. Materials and Methods

### 2.1. Reagents

All chemicals were of analytical grade and used as received without any further purification. Solutions were prepared with MilliQ water (Millipore, Inc.; Ω = 18 MΩ/cm). L-ascorbic acid (99%) was purchased from Merck (Germany); stock solutions of AA were prepared daily in phosphate buffer (PBS) at pH 6.2. The phosphate buffer saline solution was made using NaCl (137 mM), KCl (2.7 mM), Na_2_HPO_4_ (8.1 mM) and KH_2_PO_4_ (1.47 mM) from Sigma and then adjusted to pH 6.2, which is both the measured pH of parsley and the optimum for enzymatic activity of ascorbate peroxidase, which is in the range of 5.5 to 7.0. Ascorbate oxidase from *Cucurbita* sp. (EC 1.10.3.3) was purchased from Merck; one unit oxidizes 1.0 μmol of L-ascorbate to dehydroascorbate per min at pH 5.6 at 25 °C.

### 2.2. Parsley Samples Preparation

Parsley samples, fresh and fresh-cut (chopped), were collected by Fresco & Pronto S.r.l. (Selargius, CA, Sardinia, Italy), a company specialized in the production of minimally processed fruit and vegetables. Fresh-cut parsley is processed following HACCP guidelines [36] and packaged in 80 g see-through resealable polypropylene trays for foodstuffs (Figure S1 in Supplementary Materials) that, immediately after processing (day 0), are moved to a sorting center by refrigerated vans. From there, early the next day (day 1), they are sorted to the stores, where they are sold with an expiry date of six days from processing.

### 2.3. Experimental Protocol

In accordance with the company protocol, this experimental plan provided for the in situ (on company premises and in transport vans) monitoring of AA in parsley juice for six days (from day 0 to day 5). AA content was recorded before and immediately after processing (day 0) and daily until the expiration date (day 5). The following experimental treatments were compared:

(1) FP-Ctrl—Fresh parsley (not processed) properly stored at about 6 °C, used as control;
(2) FCP—Fresh-cut (chopped) parsley properly stored at about 6 °C. At day 0, AA content was measured immediately after chopping;
(3) FCP-int—Fresh-cut parsley stored at about 6 °C, but with an interruption of the cold chain. Trays were stored at 28 °C for 2 h, immediately after processing (day 0), in order to simulate a cold chain interruption during a road transport to sorting center. The average atmospheric temperature in July in southern Sardinia is 28 °C. At day 0, AA content was measured immediately after 2 h at 28 °C;
(4) FCP-frz—fresh-cut parsley frozen at −2 °C for 2 h immediately after processing and then gradually brought back to the storage temperature of 6 °C, in order to simulate an unexpected failure of the thermostat of the van and a consequent drop in temperature below zero degrees centigrade. At day 0, AA content was measured immediately after the 2 h at −2 °C.

The juice for analyses was obtained by a cold extraction from 80 g of parsley, fresh or fresh-cut, using a DAYA juice extractor for home appliances (power 200 W/centrifuge 60 rpm) (Consumer Electronics S.p.A., Legnano, Italy). 

### 2.4. The Ascorbate Bluetooth© Analyzer

A detailed description of the amperometric (Figure S2 in Supplementary Materials) and telemetry (Figure S3 in Supplementary Materials) modules, as well as all the specifications about the firmware and Software of the Ascorbate Bluetooth© Analyzer are reported in Supplementary Materials.

### 2.5. Sensors Description, Characterization and Calibration

**Figure 1:**
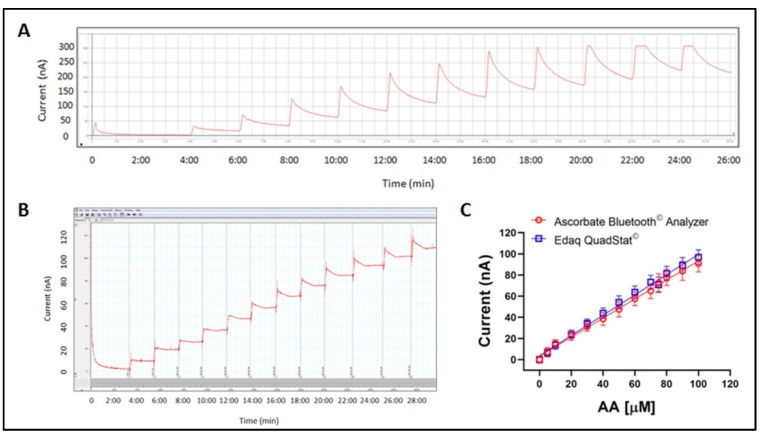
In vitro calibration of AA performed with ABA (**A**) and Quadstat (**B**), respectively. AA regression lines with the two systems starting from 0 up to 100 μM (**C**). Values are means ± standard deviation, *n* = 4.

The screen-printed sensors used in this work were purchased by GSI Technologies (311 Shore Drive, Burr Ridge, IL, USA—www.GSITech.com, accessed on 5 February 2019) and consist of a 5 mm carbon working electrode (WE), an Ag/AgCl pseudo reference electrode (RE) and a carbon auxiliary electrode (AE) (Figure 1). More details are reported in Figure S4 in Supplementary Materials.

AA oxidation on the WE surface was studied in order to set the working potential. Cyclic voltammograms (CV) of increasing AA concentration (from 1 mM to 4 mM) were performed from −1 V to +1 V (vs. Ag/AgCl pseudo-RE) at a scan rate of 0.1 V/s.

AA calibrations were carried out by constant potential amperometry, both by ABA and Quadstat, a commercial four-channel potentiostat (eDaQ Quadstat, e-Corder 410 and Echem software, eDAQ Europe Poland) as follows: a first aliquot of 70 µL, containing only PBS (used as supporting electrolyte), was deposited on the sensor surface with a graduated micropipette in order to obtain a baseline. A positive potential of +120 mV was applied vs. Ag/AgCl pseudo-RE. Once the baseline current was recorded, the PBS drop was dried with absorbent paper without touching the surface of the sensor. Subsequent 70 µL aliquots of increasing AA concentrations (from 0 to 100 µM) were deposited on, and removed from, the sensor surface by the same technique. The current values were read out every two minutes.

Chronoamperometric experiments were carried out according to [37] in order to analyze the decay of the current response and estimate the diffusion coefficient of AA.

### 2.6. AA Electrochemical Detection in Parsley

The electrochemical detection of AA was performed by ABA immediately after juice extraction, and data were compared to those obtained by Quadstat. The SPEs used in this work did not require any activation pretreatment before use. AA currents (nA) were obtained by simply exposing the screen-printed sensors surface to 70 µL of diluted (1:10 in PBS) parsley juice (Figure S5 in Supplementary Materials).

### 2.7. Kinetics Analysis

In order to understand which mathematical model best fit the AA decay in fresh and fresh-cut parsley, data were analyzed using the available linear and non-linear models of the statistical software GraphPad Prism 5 for Windows software (GraphPad Software, Inc., La Jolla, CA 92037, USA). Data fitting was considered to be significant at a probability level of 95%. The models chosen as the most representative are described in the results.

### 2.8. Determination of Protein Content and Activity of AA Oxidative Enzymes

#### 2.8.1. Sample Preparation

Proteins were extracted from parsley samples with cold PBS without protein inhibitors at 4 °C. Two grams of fresh cut parsley were transferred in a 15 mL round-bottom tube, and 2 mL of cold PBS were added. Samples were homogenized by an Ultra-Turrax device, two times for 30 s, and then they were centrifuged for 15 min at 4 °C at 6000 rpm. The supernatant was transferred to 2 mL tubes and stored at −20 °C. Protein content was determined by Qubit Protein Assay Kit in accordance with the protocol provided with the kit.

Ponceau staining was performed to confirm that the amount of protein was identical in all samples (Figure S6 in Supplementary Materials). A total of 50 µg of total protein was separated on 10% SDS-PAGE following a standard protocol (Biorad, Segrate, Italy). Samples were electroblotted onto Protran premium nitrocellulose membranes (Thermo Fisher Scientific, Rodano (MI), Italy), stained with a 0.1% Ponceau solution and then washed twice in ultrapure water for 3 min. A total of 5 U of AOx from *Cucurbita* sp. was loaded as positive control for AOx activity.

#### 2.8.2. Ascorbate Oxidase Activity

*Native gel electrophoresis*. As similarly reported for superoxide dismutase 2 activity [38], 50 µg of total protein and 5 U of ascorbate oxidase (AOx) from *Cucurbita* sp. (Merck Life Science, Milan, Italy), used as positive control, were subjected to electrophoresis under non-denaturing, non-reducing conditions at 40 mA per gel for 5 h at 4 °C. A 10% polyacrylamide gel was used and stained with nitroblue tetrazolium reagent (NBT).

*Native gel staining*. Native gel was stained with NBT as described by [39] with some modifications. The gel was treated with 0.1 M H_2_O_2_ for 10 min and washed twice in H2PO4 buffer for 10 min. The gel was stained in the dark by immersion in a 0.1 mM NBT solution for one hour and in a solution containing 36 mM H_2_PO_4_, 28 mM Temed and 35 µM riboflavin, for another hour. 

The gel was then put under a white light for 16 h at 15 °C until a clear band appeared. The reaction was stopped by rinsing the gel in ddH_2_O.

#### 2.8.3. Ascorbate Peroxidase Activity

Ascorbate peroxidase (APx) activity was determined according to [40] with some modification: 1 mL buffer, containing 0.2 M Tris-HCl pH 7.8, 1 mM ascorbic acid and 2.5 mM H_2_O_2_, was prepared for 50 µL of sample. The procedure was carried out at 4 °C. The reduction of absorbance was measured at 290 nm for 1 min at 25 °C. 

##### Western Blotting

Fifty micrograms of total protein were separated on SDS-PAGE following standard protocols (Biorad, Segrate, Italy). Samples were electroblotted onto Protran premium nitrocellulose membranes (Thermo Fisher Scientific, Rodano (MI), Italy). After 1 h of 5% low-fat milk in PBS-Tween 0.01% solution (blocking solution) incubation, membranes were incubated with 5% low-fat milk in PBS-Tween 0.01% solution with the indicated antibody for 16 h at 4 °C. Anti-Rabbit IgG (whole molecule)-peroxidase antibodies were used to reveal immunocomplexes by enhanced chemiluminescence. Total ascorbate peroxidase (1:2000) was the antibody used for Western blotting. LiteAblot Plus Enhanced Chemiluminescent Substrate system (Euroclone, Milan, Italy) was used for detection. Blots were quantified using the Image Studio Lite densitometry program (LI-COR, Lincoln, NE, USA).

### 2.9. Statistical Analysis

Statistical analysis was performed by GraphPad Prism 5 for Windows software (GraphPad Software, Inc., La Jolla, CA 92037, USA).

AA currents obtained by SPEs were expressed in nanoamperes and given as mean ± standard deviation (SD) (*n* = 4) of absolute oxidation currents (nA) or baseline-subtracted currents (DnA). After in vitro calibrations, the AA currents were plotted vs. the AA concentration, and the linear regression was calculated. AA juice content was expressed as molarity and then converted to mg/100 g, in the form usually used on food packaging. In order to assure the similarity between data obtained by ABA and Quadstat, a Student’s *t*-test to compare means was performed. Differences among treatments were compared by ANOVA using a unifactorial complete randomized block design. Mean comparisons were calculated by Fisher’s least significant difference test (LSD) at *p* ≤ 0.05.

*Ascorbate oxidase activity*. ImageJ software was used to measure the optical density (OD) (densitometric analysis) of the AOx activity, expressed as AOx enzyme units. The AOx enzyme units were calculated normalizing the OD of the samples by the OD of the positive control as follows: 

(densitometry of the sample × 5 (units of the positive control))/densitometry of the positive control.

*Ascorbate peroxidase activity*. ImageJ software was used to measure the optical density (OD) (densitometric analysis) of APx protein separated with the Western blot technique. The APx activity, expressed as µmol of AA converted by APx, was calculated first by dividing the absorbance of the sample to the absorbance of the positive control containing 1 µmol of AA, then by normalizing the obtained value by the quantity of the protein in 50 µL.

## 3. Results

### 3.1. The Ascorbate Bluetooth© Analyzer and Calibration of Screen-Printed Sensors

ABA was tested using a Thevenin current generator as illustrated in previous studies [41,42]. When powered by battery, the noise was limited to 25 pA while it increased about three times when the circuit based on the TP4056 chip was connected (for battery charging). This aspect, already foreseen in the design phase, confirmed the usefulness of battery power on noise and, during all the sensor calibration and sample analysis experiments, the device was always powered by the battery by disconnecting the charging circuit by means of the appropriate switch (see object 8 in Figure S3 in Supplementary Materials). All experiments were conducted within a linear distance of 10 m and without infrastructural obstacles placed between the device and the personal computer used for monitoring and recording digital signals. In these conditions, the selected sample rate (5 Hz) guaranteed error-free transmission during the entire period of use. 

A series of experiments was carried out by calibrating screen-printed sensors, a first group connected to the ABA and a second group connected to the Quadstat, with the same ascorbic acid solution. The images of AA calibrations with ABA and the Quadstat are reported in Figure 1A,B, respectively. Four screen printed sensors were connected in succession to each analytical system and calibrated in parallel in order to demonstrate the repeatability of measures taken by screen-printed sensors and to compare the performance of ABA with the data obtained with the Quadstat, which was already tested and had proven reliability [13,14,17].

The screen-printed sensors provided linear calibration with a r^2^ = 0.996 and 0.994 with ABA and Quadstat, respectively, in the tested concentration range, thus demonstrating that there was no difference between estimated and measured current values (Figure 1C). According to the *t*-test, the slope values were not significantly different (0.8997 vs. 0.9570 for ABA and Quadstat, respectively) revealing that, compared to the more sophisticated and expensive Quadstat, the sensors combined with the ABA have the same sensitivity to variations in AA concentration.

### 3.2. Characterization, Specificity, Limit of Detection (LOD) and Aging of Screen-Printed Sensors

Before starting AA measurements in parsley, the specificity of the system towards AA was demonstrated to eliminate the possibility that the recorded currents were influenced by interferers. On the basis of the performed cyclic voltammograms (Figure S7 in Supplementary Materials) and in accordance with previous results [13,17], the applied potential was set at +120 mV vs. Ag/AgCl pseudo-RE. This working potential is sufficiently high for a correct reading of the AA and low enough to exclude a large number of molecules that could influence the recorded currents in parsley juice. About 30 polyphenols were identified in parsley [43], all having different redox potentials, but not all of them could be potential interferers. According to [44], the glucoside forms of apigenin accounted for more than 98% of the total polyphenols in parsley; these molecules have low antioxidant capacity since they start to be oxidized at about +800 mV [45] and cannot be recorded at the potential we applied to the ABA. Among the other quantified flavonoids [44], only the quercetin-3-O-galactoside started to be oxidized at a potential close to AA (Figure S8 in Supplementary Materials), but it was represented in an extremely low quantity compared to AA (<0.5 mg/100 g vs. 120 mg/100 g FW, respectively), so that the interference on the recorded AA currents was absolutely negligible. Moreover, all the identified hydroxycinnamic acids, caffeic, chlorogenic, coumaric and ferulic acids started to be oxidized at a potential higher than +250 mV [14], while other molecules like sugars or organic acids could be excluded from the list of possible interferers according to previous research [13].

The LOD of the sensor was 1.38 ± 0.13 µM, calculated as 3.3 *σ/S*, where *σ* is the standard deviation of background noise of the screen-printed sensor, and *S* is the slope of the linear region of the calibration curve, which means that ABA could be used for samples containing at least 2.4 mg AA/100 g FW. 

The stability of the measurements of the screen-printed sensors is a false problem, because they are sold in multi-sensors sheets, at a limited price, printed to be used only once. However, an estimate of the aging of the sensors and the reproducibility of the measurements was carried out, both with subsequent calibrations of standard AA and with 10 subsequent injections of 70 µL of parsley juice, rinsing the sensor at each measurement. In the former case, the slope values of the regression line decreased from 0.8869 to 0.2654 after 11 calibrations. In the second case, however, from the first to the fifth measure, a loss of about 25% of the initial values was observed, then from the sixth to the tenth measure, the recorded currents dropped by 70% compared to the initial ones.

#### Chronoamperometry

**Figure 2:**
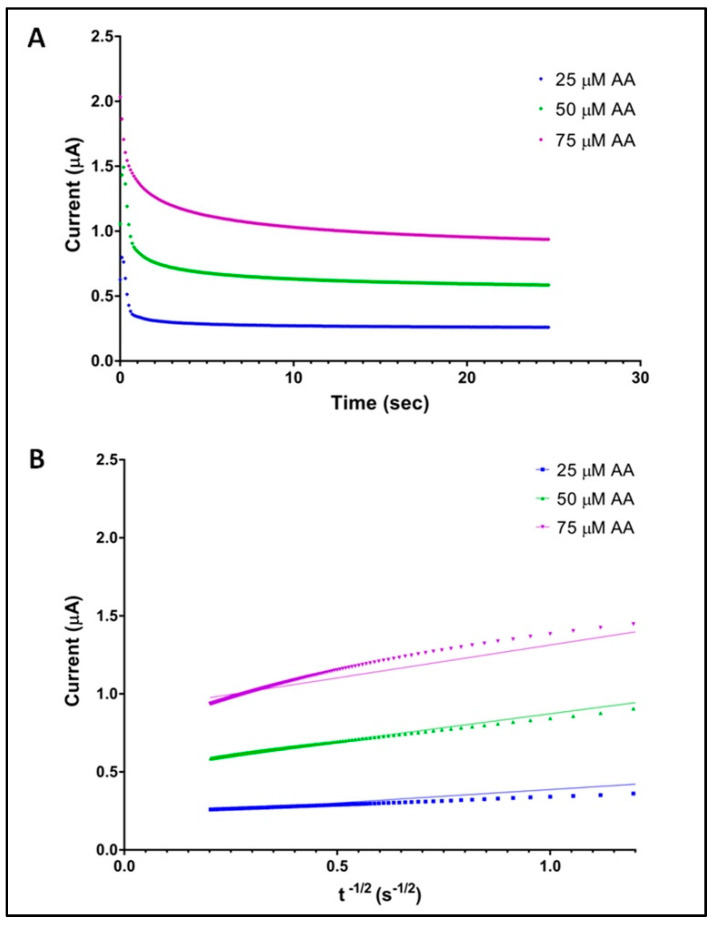
Chronoamperometries of SPE in PBS for different concentrations of ascorbic acid (**A**). Cottrell plots of *I* vs. t^−1/2^ (s^−1/2^) of the chronoamperometric data obtained for 25, 50 and 75 uM of AA (**B**).

The chronoamperometric analysis for AA was performed by SPE vs. Ag/AgCl pseudo-RE at +120 mV. Chronoamperometric results obtained for different AA concentrations in PBS (pH 6.2) are shown in Figure 2A.

The Cottrell equation for chronoamperometric analysis of electroactive moieties under mass transfer limited conditions is

*I* = *nFAD*^1/2^*cπ*^−1/2^*t*^−1/2^

where *n* is the number of electrons involved in the rate determining step (*n* = 2 for AA); *F* is the Faraday constant of 96,485 C mol^−1^; *A* is the geometric area of WE of SPE (0.19547 cm^2^); *D* is the diffusion coefficient; *c* is the [AA] and *t* is the time (s). According to [37] this equation can be used to estimate the diffusion coefficient of AA. The slope of the linear region of the *I* vs. *t*^−1/2^ (Figure 2B) in the short time region provides the product AD^1/2^, so *D* was calculated as 3.01 × 10^−12^ cm^2^ s^−1^.

### 3.3. AA Monitoring in Parsley

**Figure 3:**
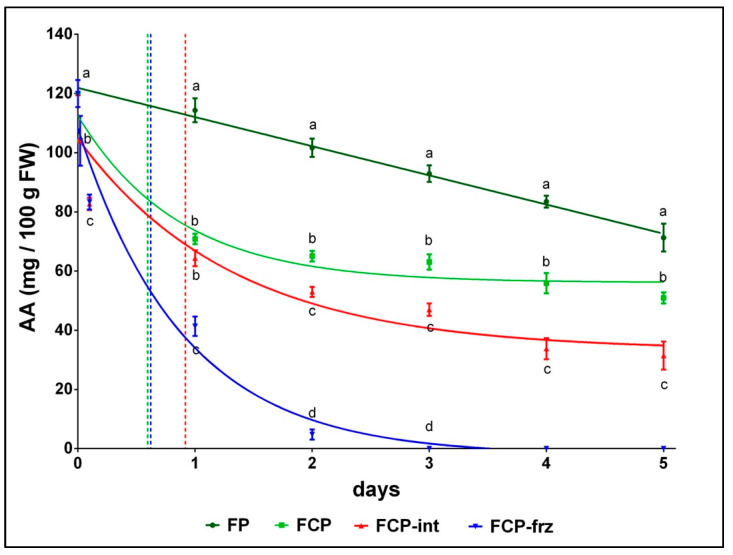
AA kinetics decay in fresh and fresh-cut parsley under different storage conditions, detected with ABA. Values are means ± standard deviation, *n* = 3. Within each storage time, means followed by unlike letters differ significantly by Fisher’s least significant difference (LSD) test, *p* ≤ 0.05. Light green, red and blue vertical lines intersect the FCP, FCP-int and FCP-frz curves at their respective half-lives.

ABA was successfully used for AA detection on fresh and fresh-cut parsley, and the results of monitoring are reported in Figure 3. The mathematical models that best represented the AA decay were different depending on processing and storage temperatures: the linear regression model for FP, and the one-phase decay model for FCP, FCP-int and FCP-frz.

AA decay was linear in fresh parsley with a daily loss between 5% and 10% until the end of storage, −40% at day 5; *y* = −9.854 *x* + 121.9 is the equation of the regression line that best represented this trend, where *y* is the AA level (mg/100 g FW) at any time *x*, with a coefficient of determination r^2^ = 0.993. 

At day 0, the effects of processing (FCP) resulted in a 15% AA drop vs. FP-ctrl, detected immediately after chopping. Two hours of exposure at 28 °C (FCP-int), as well as at −2 °C (FCP-frz), resulted in a further 20% AA decrease compared to FCP. Then, restoring proper storage temperatures had different consequences that can be summarized as follows:

- The parsley processing determined a rapid decay of AA according to the following equation:
  AA_t_ = (AA_0_ − plateau) _*_ exp(−k_*_x) + plateau
  where AA_t_ is the AA content at any time *x* (day 0, day 1 … day 5), AA_0_ is the initial value of untreated samples, k is the rate constant, expressed in reciprocal of the x time units, and plateau is the AA value at infinite times, expressed in the same units as AA.
- Chopping determined a rapid decay of AA followed by a gradual decline to a plateau in FCP and FCP-int. Although the r^2^ of the curves were 0.9362 and 0.9070, respectively, among all the tested models, the one-phase decay was the one best suited to these two experimental groups. When the cold chain was maintained (FCP), the decay was rapid for about 14–15 h (half-life = 0.5957), and a plateau was reached at 56.12 mg/100 g of AA. On the other hand, as a consequence of 2 h at + 28 °C, the rapid decay lasted longer, about 22 h (half-life = 0.9184), and the plateau was reached only after consuming almost the 70% of the initial AA (at 33.24 mg/100 g of AA).
- The one-phase decay model also best described the AA decay in FCP-frz samples (r^2^ = 0.9741). The half-life value of AA decay curve samples was 0.6216, thus indicating that two hours freezing resulted in a 50% AA consumption in the first 15 h, similarly to FCP. However, the gradually thawing up to the correct storage temperature had a dramatic effect on the AA content, which dropped by 60% after 24 h and another 35% over the next 24 h.

### 3.4. Enzymatic Activity in Parsley Samples

Kinetic analysis showed significant differences in differently treated parsley samples. Supported by the literature [46], we investigated the role of enzymatic systems that use ascorbate as a substrate to understand if, how much and how AOx and APx are responsible for the reduction of the AA content in fresh and fresh-cut parsley. 

**Figure 4:**
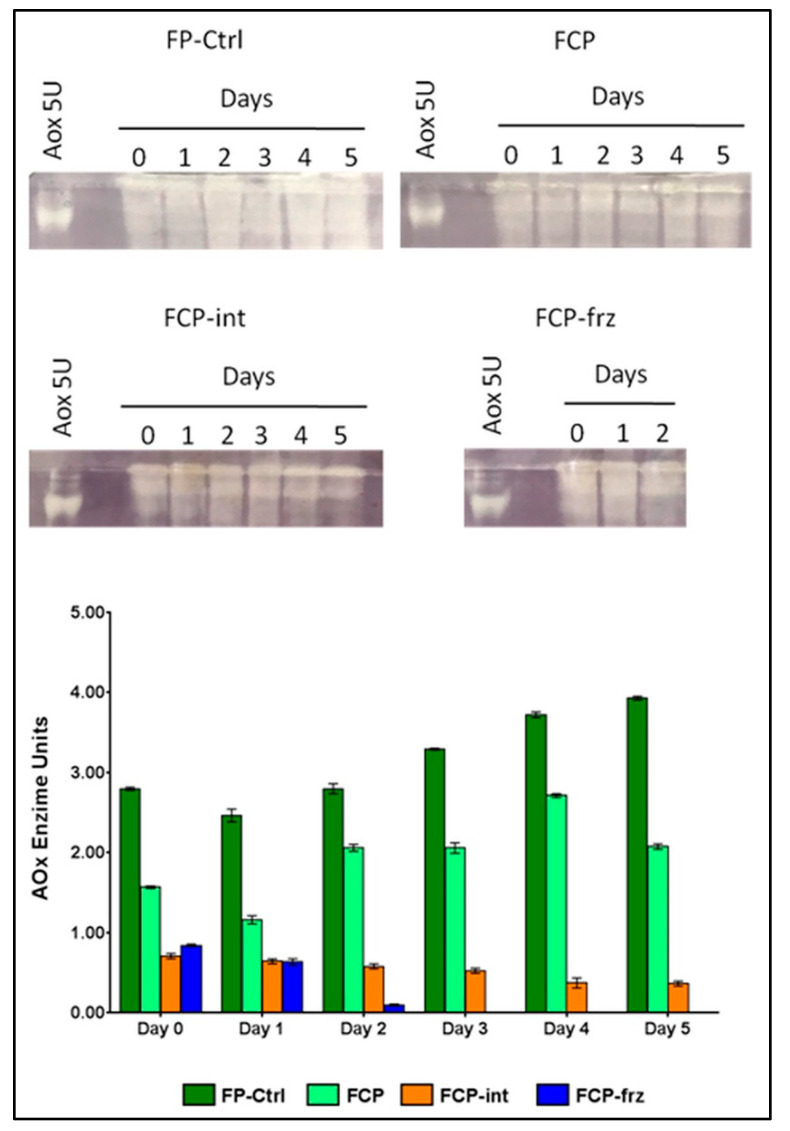
Changes in AOx activity in fresh and fresh-cut parsley under different storage conditions, determined by Native gel electrophoresis (cropped images) and expressed as AOx enzyme units (graph). A total of 5 U of AOx was used as Ctrl (1 unit oxidizes 1.0 μmol of L-ascorbate to dehydroascorbate per min at pH 5.6 at 25 °C). Values are means ± standard deviation, *n* = 3. Mean comparisons are reported in Table S1 in Supplementary Materials.

The results of AOx activity in fresh and fresh-cut parsley are presented in Figure 4 (see also the full-length uncropped gel picture as Figure S9 in Supplementary Materials).

The graph shows that AOx activity was higher in fresh than in fresh-cut parsley, almost double in FP than in FCP. When the cold chain was respected, as in FP and FCP, we saw a slight decrease in activity on day 1 and a progressive increase afterwards, up to day 5 in FP and up to day 4 for FCP. In the latter, a slight decline was observed on day 5, although activity was still significantly higher than on day 0.

The effects of the interruption of the correct operating temperature were statistically similar, up to 24 h from such a change, both in FCP-int and in FCP-frz; in both cases there was a reduction of about 50% of the activity of the AOx vs. FCP. However, when the cold chain was restored, the AOx activity in the FCP-int remained constant throughout the shelf-life, while it inexorably collapsed in the FCO-frz.

**Figure 5:**
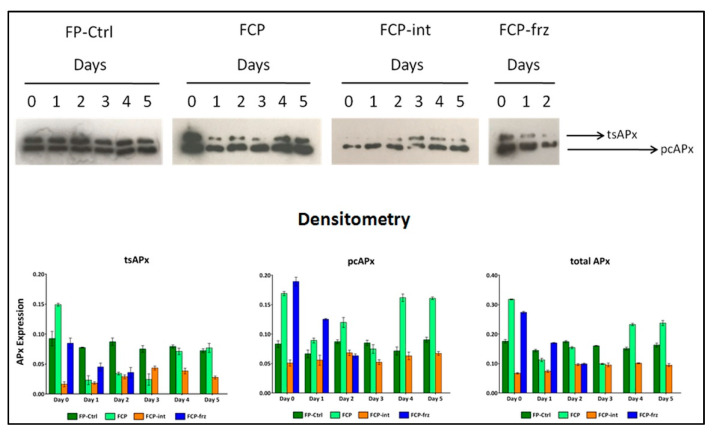
Changes in APx expression in fresh and fresh-cut parsley under different storage conditions, determined by Western blot analysis (cropped images), from three independent experiments and normalized with Ponceau (see Figure S6 in Supplementary Materials). The expression of thylakoid and stromal (tsAPx), peroxisome and cytosolic (pcAPx) isoforms, as well as the total APx, is shown in the graphs according to the optical density of APx protein. Values are means ± standard deviation, *n* = 3. Mean comparisons are reported in Table S2 in Supplementary Materials.

The Western blot images in Figure 5 (see also the full-length uncropped gel picture as Figure S10 in Supplementary Materials) show the expression of thylakoids and stromal (tsAPx), as well as peroxisomes and cytosolic (pcAPx) APx isoforms, in fresh and fresh-cut parsley under the tested storage conditions.

Densitometry revealed that the total expression of the enzyme remained almost unchanged over the days in FP. In FCP, when the cold chain was properly maintained, total APx expression rose as a result of chopping, fell over the next 72 h and returned to significantly higher levels of FP on days 4 and 5. Two hours at 28 °C (FCP-int) determined an immediate significant decrease in the total APx expression compared to the FCP. In FCP-frz, the frozen sample showed an expression of the enzyme equal to FCP, but the thawing and the restoration of the operating temperatures induced a reduction of the expression of 40% every 24 h. Similar considerations could also be extrapolated from the densitometric analysis of the expression of the different isoforms. The pattern of APx expression in chloroplasts (tsAPx) was similar to that determined in peroxisomes and cytosol (pcAPx), but in the latter the expression was higher.

The difference in APx activity among treatments is shown in Table 1. No significant changes in APx activity were observed in fresh parsley until day 3; then there was a sharp decrease on day 4 to a level that remained low on day 5 as well. Immediately after chopping, the APx activity remained unchanged from the control, but a progressive and significant increase was monitored in the next 48 h. A new decrease to lower values than the initial ones was then observed. In FCP-int, the heat shock caused an immediate increase in APx activity compared to FCP, and a progressive increase in the same in the following days. On day 5, the activity values were even higher than those of the FCP. At last, the APx activity in FCP-frz was significantly higher than the other treatments at day 0. It decreased as the optimal storage temperature was restored but remained high on day 2 and day 3.

## 4. Discussion

Fresh-cut fruit and vegetables deteriorate faster than the unprocessed raw materials, mainly due to the damages caused by minimal processing techniques. Processing operations directly shorten the shelf life of fresh-cut produce and indirectly by softening and browning tissues, decreasing nutritional value, developing off-odors [47,48] and exposing cut surfaces to pathogenic microorganisms [7,8]. Shelf-life extension has been the main goal towards fresh-cut produce since they were first proposed to consumers, while quality monitoring has often been subordinated to compliance with health standards. Having placed the health of the consumer in the foreground, also the indicators of correct conservation were mainly dedicated to the control of the pathogenic microbial load [8] or residuals of sanitization [49]. Although the literature reports many articles on nutritional properties and on the inevitable qualitative decay, both in optimal and non-optimal storage conditions, in the real supply chain there is no control of the nutritional characteristics of fresh-cut produce, except that “flavor and odor must be acceptable for the consumer without anomalous variations” (HACCP Manual “Reg. CE 2073/05” and “DM no. 3746—20 of June 2014”). The load of pathogenic microorganisms requires sophisticated laboratory analyzes, while flaws of visual quality and off-odors are manifested only late when the produce is inevitably deteriorated [50]. There are no monitoring systems based on quality indicators, on the fresh-cut market, that report in real time the effects of inefficiency or malfunction in the cold chain and that allow operators to intervene and remedy immediately.

For this reason, telemetry was considered, from the initial design stages, as a key element of this study, and the choice of Bluetooth© technology is justified by the ubiquitous diffusion of this standard and by the high quality and low cost of the interfaceable modules available on the market. Telemetry allows processes to be monitored at a distance and in inaccessible or potentially dangerous environments. Furthermore, it considerably increases the signal-to-noise (S/N) ratio, mainly due to the absence of a wired connection that generally injects noise in the absence of appropriate insulation, which is often complex to design and which increases the cost of the device. Furthermore, due to the electronic power supply, it is necessary to isolate the analyzer from the personal computer (or smartphone) to avoid the injection into the digital systems of voltage spikes that could damage the delicate electronic components. Coupling the electrochemical detector for ascorbic acid with Bluetooth© transmission is easy, since screen-printed sensors (SPEs) enable simple integration and the portability needed for on-field applications [30]. The SPEs used in this work provide robustness and repeatability of the measurements compared to pencil leads whose main flaw, which limited their diffusion on the market as sensors, was fragility [13,14,17]. The chronoamperometric analysis allowed for the determination of the diffusion coefficient. The D value calculated in this work was compared with other AA diffusion values on carbon electrodes. It was lower than [51,52,53] and higher than [54]. It is conceivable that these differences are attributable to the different concentrations tested, to the different materials used to functionalize the sensors and to the different applied potentials. Our low D value is compatible with low concentrations of the analyte (the system works between 0 and 100 uM), with a bare carbon electrode and at a low applied potential. This also confirmed that SPEs are a suitable tool to determine the AA concentration in parsley, although always in the same batch. This is because the system works with a pseudo-RE. This mean that comparable measurements can be recorded only if the analyzed matrices have constant or nearly constant [Cl^−^] concentrations. This can only happen if parsley samples belong to the same batch. Comparison between different batches is not possible and would not make sense.

Regardless of the technique adopted to extend the shelf-life, this work focused on the characteristics of a monitoring system capable of alerting the producers or sellers, or anyone else, of small variations in the AA content caused by unexpected interruptions in the cold chain. This is because it has been demonstrated that the AA content is a reliable indicator of the quality of fruit and vegetables and that its decay corresponds to a decline in the nutritional values of these produce [10,12,24,55]. Our results demonstrated that the first 24 h from processing constitute the most delicate phase of the parsley supply chain, when the produce is moved from the company to the sorting center and from there to the points of sale. It is in this time interval that an interruption of the cold chain is more likely, and it is in this time interval that we simulated a heat and a cold shock. 

Kinetics modeling has been successfully used to predict the influence of processing and storage on AA variation in juice [46]. Our results indicated that the AA content of chopped parsley, and consequently its quality, undergoes a rapid decay due to the processing effect alone, even in the best storage conditions, while an interruption of the cold chain at 28 °C for 2 two hours results in a much faster and significantly higher consumption of AA, in accordance with previous studies on fresh-cut kiwi and pineapple [17] or spinach [18]. The immediate restoration of the optimal storage temperatures allows the matrix to limit the qualitative decay, and the rapid consumption of AA is followed by a gradual decline to a plateau whether the processing takes place in full compliance with the cold chain (FCP) or if there is a rise in temperature immediately after chopping (FCP-int). The consequences of the cold stroke, on the other hand, were unexpected because the visual investigation following a two-hour freezing (−2 °C)/thawing cycle did not foreshadow the very strong impact on cellular tissues that certainly occurred. The visual appearance (Figure S11 in Supplementary Materials) and smell of FCP and FCP-frz parsley after 24 h were the same, but the AA content was totally different.

The curves of AA decay are reliable indicators of the combined action of oxidative enzymatic activity and ROS, since fresh-cut processing resulted in a loss of cellular compartmentation at the cut surface and as a consequent an increase in interaction between AA oxidative enzymes and their substrate [56]. The hypothesis was that free radicals and H_2_O_2_ acted together with enzymatic systems to degrade AA. The fact that AA decay in parsley followed different patterns according to different treatments corroborated this assumption. When the AA decay is linear (FP) the AOx activity is high and increases linearly over the days. Chopping reduces AOx activity in parsley, although conflicting data are found in the literature, as AOx expression has been shown to be induced [21] or repressed [20] by wounding. When the cold chain is properly maintained, the AOx activity increases, probably because the plant’s defense systems remain intact for longer at low temperatures. Obviously, in chopped parsley, the positive effect of low temperatures lasts less. So, in the FCP, we probably had a wounding effect that reduces, and a low temperature effect that increases, the activity of AOx. Until day 4 the second prevails; on the fifth day the first prevails. Finally, data of AOx activity in FCP-int and FCP-frz indicated that chopped parsley seems to tolerate better the effects of a heat stroke than a sudden freezing and thawing cycle that inexorably compromises its shelf-life.

The combined effects of chopping and changes of optimal temperature conditions also influenced APx expression and activity. APx is the key enzyme of the ascorbate–glutathione cycle in plant chloroplast, but it also acts as an ROS scavenger in cytosol, mitochondria and peroxisomes [57]. Its role consists of preventing the accumulation of H_2_O_2_ produced by oxidative stress, reducing it to H_2_O using the reducing capacity of AA [20,58]. In our study, APx expression did not change in fresh parsley, but it immediately increased as a result of chopping. It was previously observed that APX activity rose after cutting, particularly within the first hours after this procedure, in spinach [18], in broccoli [59] and in poplar [60]. Our results showed that immediately after chopping, the APx activity did not significantly change from control as well as the AA content. We can speculate that in the short period after processing, the plant activates the expression of APx. It is in this moment that the maximum APx expression is registered, equally in the chloroplast and cytosol. The APx, on the other hand, requires a few hours to become fully operational, and it is in the next 48–72 h that the enzyme activity increases in parallel to a significant consumption of AA.

However, the combined effect of wounding and variations in temperature strongly affected AA metabolism. APx activity strongly increased after the heat and the cold stroke consistently with the consumption of AA. In FCP-int, when the cold chain is interrupted, the relevant increase in APx activity seems to be due to the lack of effect of low temperature, in agreement with the studies of [61]. In that moment, in terms of quality, the damage is probably tremendous, and with the restoration of the right operating temperature, it is only minimized. The consumption of AA is higher than the FCP and also the activity of the APx, at least as long as there is enough AA to use as a substrate. It has been shown that when AA falls below a certain threshold, APx activity is rapidly lost [62]. This may explain the decline in APx activity observed in the last two days of storage in FCP-int.

Extreme temperatures greatly affect the quality of cultivated plants, and ROS levels tend to increase if plants are subjected to severe stress [57]. In species such as parsley, where chopping produces extreme damage, the very high APx expression observed in FCP-frz suggests that ROS production is enormous if cold damage is added to the wounding. This is in agreement with studies on potato tubers reporting that APx expression is induced more by low temperatures than by high temperatures [63]. In our work, the combined effect of the mechanical damage suffered by cells following the 2 h freezing/thawing cycle, with the enormous consumption of AA, partly lost, partly acting directly against ROS and indirectly as a substrate for APx, reduced the shelf life of fresh-cut parsley to just three days.

## 5. Conclusions

This work, and other work previous to this, indicated that the more AA there is in a species, the more the plant activates systems that use this molecule to defend itself against heat, cold or other stresses such as cutting. For this reason, the speed of AA consumption is an excellent indicator of the speed of parsley deterioration. ABA, with a very simple and feasible analysis by non-specialized operators, recorded the decrease in AA in real time and at any time along the fresh-cut parsley supply chain. This can be done on other species too, as long as they contain adequate amounts of AA. When the system is calibrated, the comparison with the initial data gives a very clear output and allows us to understand whether the optimal storage conditions have been altered or not. Obviously, it must be calibrated species by species. It has been clearly seen from previous research that microbiological load, browning and bad odors are parameters that can be detected after a period of time that is too long compared to an extremely limited shelf life. As a matter of fact, it is not possible to wait four days to remedy a problem that arose three days before, not on a six day shelf life. The AA content, on the other hand, varies almost instantly, and any storage defects, such as the interruption of the cold chain, are detectable a few hours after cutting, before, during and after transport. Such a quick analysis can remedy the problem just as quickly and help minimize the damage.

## Figures and Tables

**Table 1:** Changes in APx activity in fresh and fresh-cut parsley under different storage conditions, assayed from the decrease in absorbance at 290 nm as ascorbate was oxidized and expressed as µmol of AA converted by APx per milligram of protein per minute at 25 °C. Values are means ± standard deviation, *n* = 3. Means in columns followed by unlike letters differ significantly by Fisher’s least significant difference (LSD) test, *p* ≤ 0.05. Means in rows followed by (unlike letters) differ significantly by Fisher’s least significant difference (LSD) test, *p* ≤ 0.05.

	APx Activity (µmol mg^−1^ min^−1^)					
	Day 0	Day 1	Day 2	Day 3	Day 4	Day 5
**FP-Ctrl**	4.434 ± 0.512 c (ab)	4.784 ± 0.681 b (a)	5.114 ± 0.537 c (a)	4.199 ± 0.386 c (b)	1.211 ± 0.732 b (c)	1.197 ± 0.514 c (c)
**FCP**	4.869 ± 0.350 c (c)	7.364 ± 0.865 a (b)	10.371 ± 0.454 a (a)	6.859 ± 0.763 b (b)	7.632 ± 0.852 a (b)	3.444 ± 0.616 b (c)
**FCP-int**	6.238 ± 0.542 b (b)	6.436 ± 0.458 a (b)	7.979 ± 0.665 b (ab)	8.847 ± 0.677 a (a)	6.901 ± 0.694 a (b)	5.124 ± 0.464 a (c)
**FCP-frz**	9.288 ± 0.781 a (a)	6.717 ± 0.754 a (b)	6.888 ± 0.811 b (b)			

## Data Availability

Data supporting reported results can be found, in Italian, at the link of the project: https://www.sardegnaricerche.it/index.php?xsl=370&s=359821&v=2&c=15068&nc=1&sc=&qr=1&qp=2&fa=1&o=1&t=3&bsc=1 (accessed on 14 September 2021). Other data is contained within the article or supplementary materials.

## Supplementary Materials

The following are available online at https://www.mdpi.com/article/10.3390/antiox10091485/s1, Figure S1: Fresh-cut parsley see-through polypropylene trays at day 0; Figure S2: ABA amperometric module; Figure S3: ABA telemetry module; Figure S4: Sensor description; Figure S5: AA deposition on SPE; Figure S6; Ponceau; Figure S7: AA cyclic voltammograms; Figure S8: quercetin 3-O galactoside cyclic voltammogram; Figure S9: AOx native gel picture; Figure S10: APx western blot gel picture; Fugure S11: Fresh-cut parsley see-through polypropylene trays at day 1; Table S1: Changes in AOx activity, statistics; Table S2: Changes in APx expression, statistics.
