# Supplementary material for: An Ascorbate Bluetooth© Analyzer for Quality Control of Fresh-Cut Parsley Supply Chain
Source: Antioxidants (Basel). 2021 Sep 17;10(9):1485. doi: 10.3390/antiox10091485 (PMC8465646; doi:10.3390/antiox10091485)
Supplement: Supplementary file 1 [file antioxidants-10-01485-s001.zip › antioxidants-1355073-SI.pdf]

An Ascorbate Bluetooth© Analyzer for quality control of fresh-cut parsley supply chain

Ylenia Spissu, Antonio Barberis, Guy D'hallewin, Germano Orrù, Alessandra Scano, Gavina Rita Serra, Milo Pinna, Cristian Pinna, Salvatore Marceddu and Pier Andrea Serra

Supplementary materials

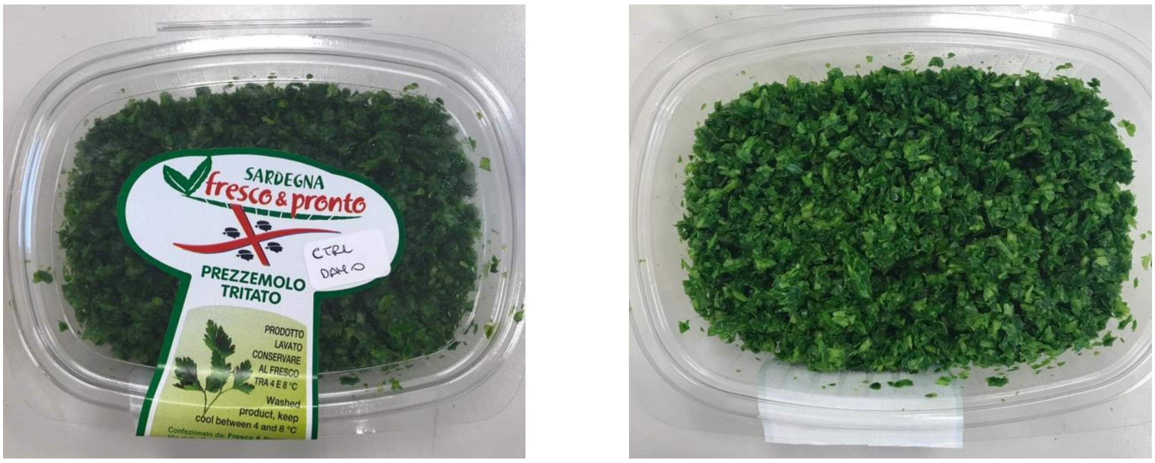

Figure S1. Fresh-cut parsley packaged in 80 g see-through resealable polypropylene trays for foodstuffs. Image taken at the company at the time of packaging (FCP at day 0).

The Ascorbate Bluetooth© Analyzer

Amperometric module of the Ascorbate Bluetooth© Analyzer

The amperometric module for the polarization of the working electrode, for the determination of ascorbic acid and for the conversion of the resulting oxidation currents (Fig. 1), was developed using a CMOS integrated circuit (Arizona Microchip MCP6044) containing 4 precision operational amplifiers (OPAs). Two OPAs were used for the construction of the potentiostat (POT), one OPA for the conversion of the AA oxidation current into a proportional voltage, and the last operational amplifier, configured as a voltage follower, was used for the generation of a buffered voltage reference (VRef). The reference (RE) and auxiliary (AE) electrodes are connected respectively to the input and output of the POT while the working electrode (WE) is connected to the input of the current-voltage converter (I / V CONV). Setting the supply voltage at 3.3V, VRef has a value of 1.65V and represents the reference potential of both the POT and the I / V CONV. The potential applied to WE was modified by acting on the POT through VApp. When VApp> VRef the system works in oxidation mode but it could work also in reduction mode (VApp <VRef). The feedback resistance of the I / V CONV has been set at a value of 10 MΩ guaranteeing a conversion factor of 100 nA / V. This aspect, associated with the fact that the MCP6044 has a rail-to-rail design, guarantees a useful current span of over 160 nA in oxidation mode and as many in reduction. The scale amplitude is compatible with the currents generated on the surface of the screen-printed electrodes used in this study. A 1 nF feedback capacitor ensures noise reduction without excessively limiting the response time of the I / V CONV. The VApp and VOut signals are connected respectively to the DAC output and the ADC input of the microcontroller unit (MCU) used in this project and described in the next section.

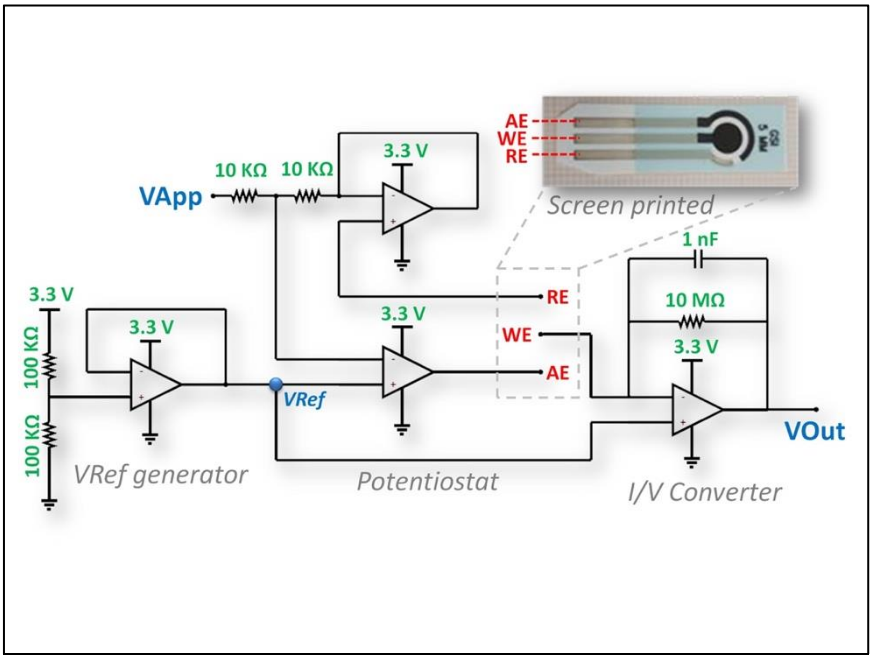

Figure S2. Schematic diagram of the amperometric module used in this project. VApp: applied voltage; VRef: reference voltage (1.65 V); VOut: output voltage; AE: auxiliary electrode; RE: reference electrode; WE: working electrode.

Telemetry module of the Ascorbate Bluetooth© Analyzer

The digital module (MCU), the telemetry module and the battery charging module together constitute the digital part of the ascorbic acid quantification device (Fig. 2). The Trinket M0 (Adafruit, New York, USA) based on a 32-bit processor (Cortex M0 + core; ATSAMD21E18) with 32 KB of RAM, 256 KB of flash memory and a clock of 48 MHz, was selected as the digital module. MCU has the task of generating the 10-bit VApp signal through the internal DAC connected to pin 1 ~ and performing the acquisition of the analog signals (VOut and VRef) through the internal 12-bit ADC connected to pins 0 and 2. The VRef signal is digitally subtracted from VOut and the serial data packet is sent to the transceiver module through pin 4. Trinket M0 is equipped with a voltage regulator and generates the 3.3V supply voltage also for the telemetry module. The pin 3 of the digital module receives telemetry data that allows to define some acquisition parameters and the sensor bias voltage (VApp). The digital module has been preloaded with a Python interpreter (CircuitPython) whose programming is started through a simple serial terminal connected to the USB port of a PC. The telemetry part of the system is based on the HC-06 module (Guangzhou HC Information Technology Co., Ltd., Guangzhou, China) which allows to convert a UART serial port into a Bluetooth© port; this device is used to allow communication between a microprocessor (MCU) and a device equipped with Bluetooth© communication, such as a PC, a Smartphone or a Tablet. The HC-06 module operates only in Slave mode: this allows the module to be used to create, for example, a network between a device containing the HC-06 module, therefore set as Slave, and a PC or smartphone set as Master. The serial transmission speed has been set to 9600 baud and the maximum transmission distance limited to 10 linear meters without obstacles. A single cell (3.7V) 1000mAh lithium polymer (LiPo) battery and a USB charger (based on the TP4056 chip from NanJing Top Power ASIC Corp., Shenzen, China) complete the circuit, ensuring 36 hours of battery life.

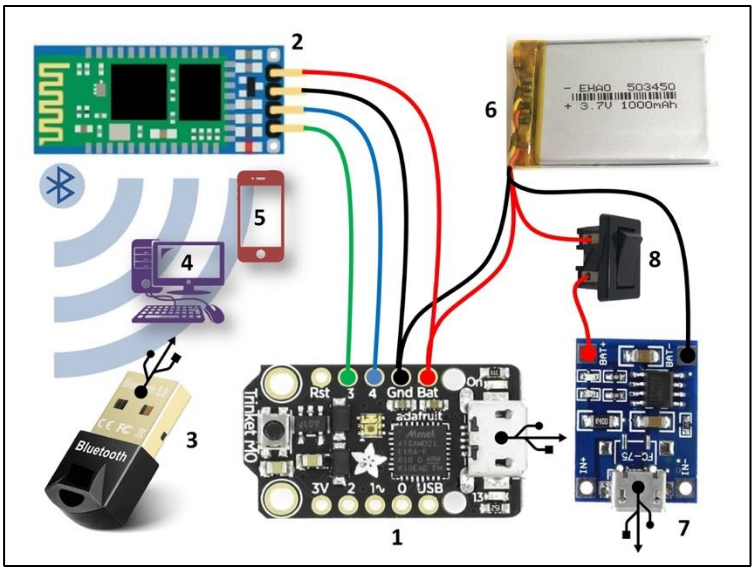

Figure S3. Schematic representation of the connections among the digital (1), the telemetry (2) and the battery charging (7) modules used in this project. The system can interface with a PC (4) equipped with a Bluetooth© interface (3) or with a smartphone (5). The USB port of the digital module allows programming of the firmware while that of the battery charger module provides the voltage needed to charge the 1000 mAh LiPo battery (6). The device was always powered by the battery by disconnecting the charging circuit by means of the appropriate switch (8).

Firmware and Software of the Ascorbic Acid Bluetooth© Analyzer

The control firmware of the Trinket M0 module was developed in CircuitPython and loaded on the digital module through a serial terminal connected to the USB port. It consists of an initialization routine of the DAC and of the ADC and of a no-blocking loop that checks a possible packet of incoming data from the Bluetooth© module and sends data packets containing VOut, VApp, VDiff (VOut-VApp) and the CRC16 for cyclic error checking. The incoming data packet allows you to set VApp and define the transmission frequency (whose standard value is set at 5 Hz). The software, which runs on the PC in a Windows environment, was developed with the Profilab Expert 4.0 development package (Abacom, Ganderkesee, Germany) and allows to receive, view, and record the data received from the device and remotely define the control potential of the AA sensor (VApp) and the sampling frequency (0.1-5 Hz).

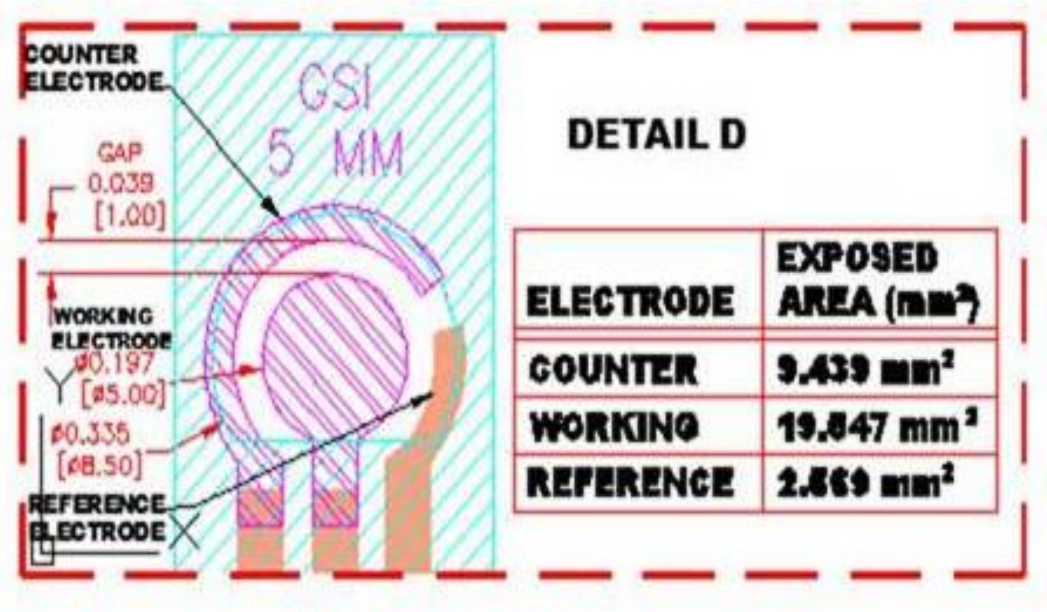

Figure S4. Sensor description with details of working, counter and reference electrodes exposed area.

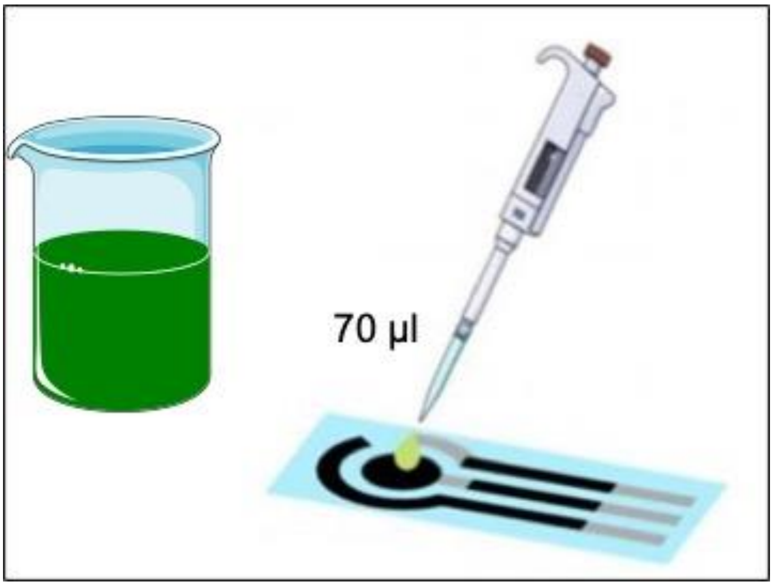

Figure S5. Deposition of 70 µl of parsley juice on the surface of the screen-printed sensor

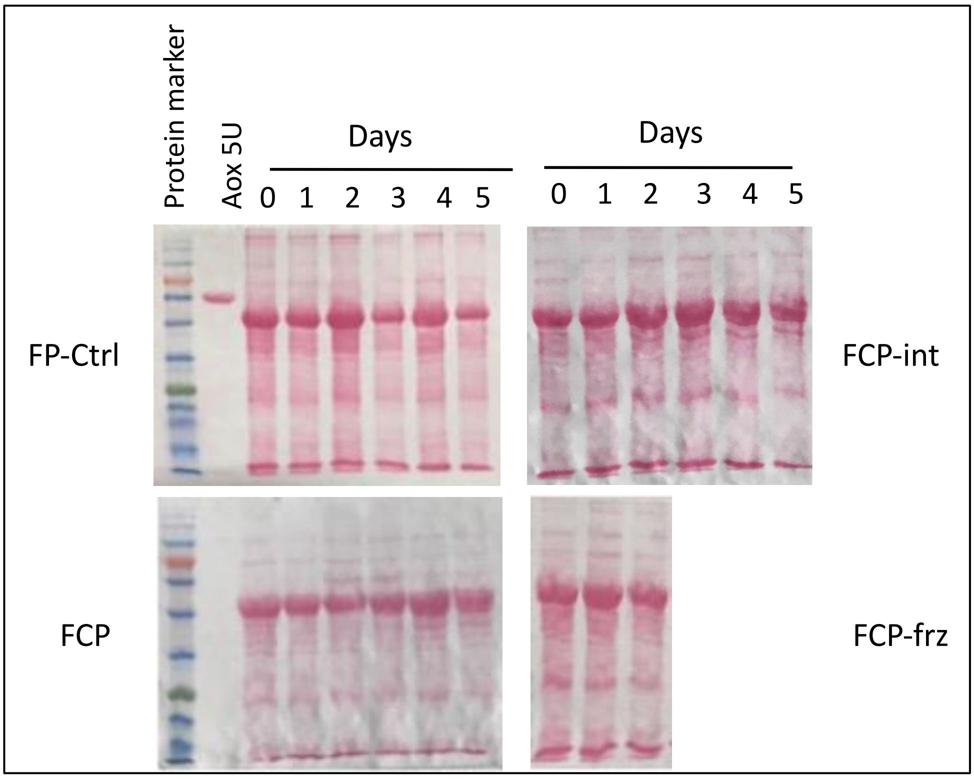

Figure S6. Electroblotted protein of parsley samples (50 µg) and ascorbate oxidase from Cucurbita sp. (5U) on nitrocellulose membrane were stained with Ponceau solution 0.01% and used to normalize data obtained from native gel (AOx activity) and western blot (APx protein quantification). A prestained protein marker (5-245 KDa) was loaded as molecular weight reference.

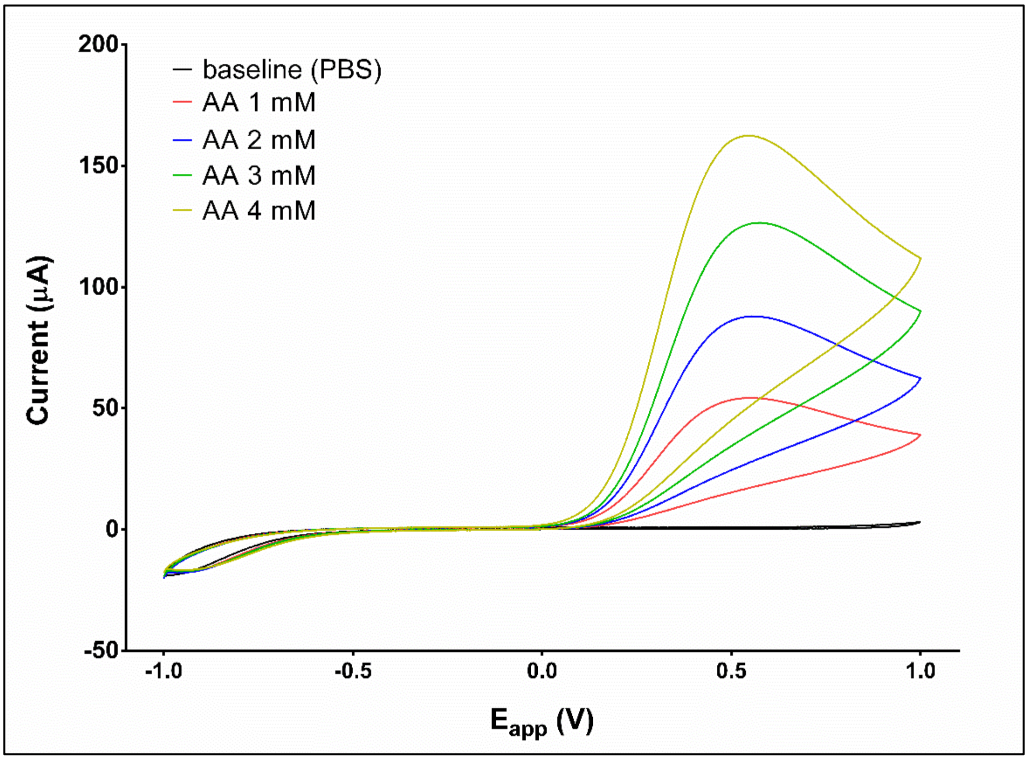

Figure S7. Cyclic voltammograms with a scanned potential range (E_{app}) comprised between -1 V and +1 V, in the absence (black line) and in the presence of 1 mM (red line), 2 mM (blue line), 3 mM green line) and 4 mM (yellow line) ascorbic acid.

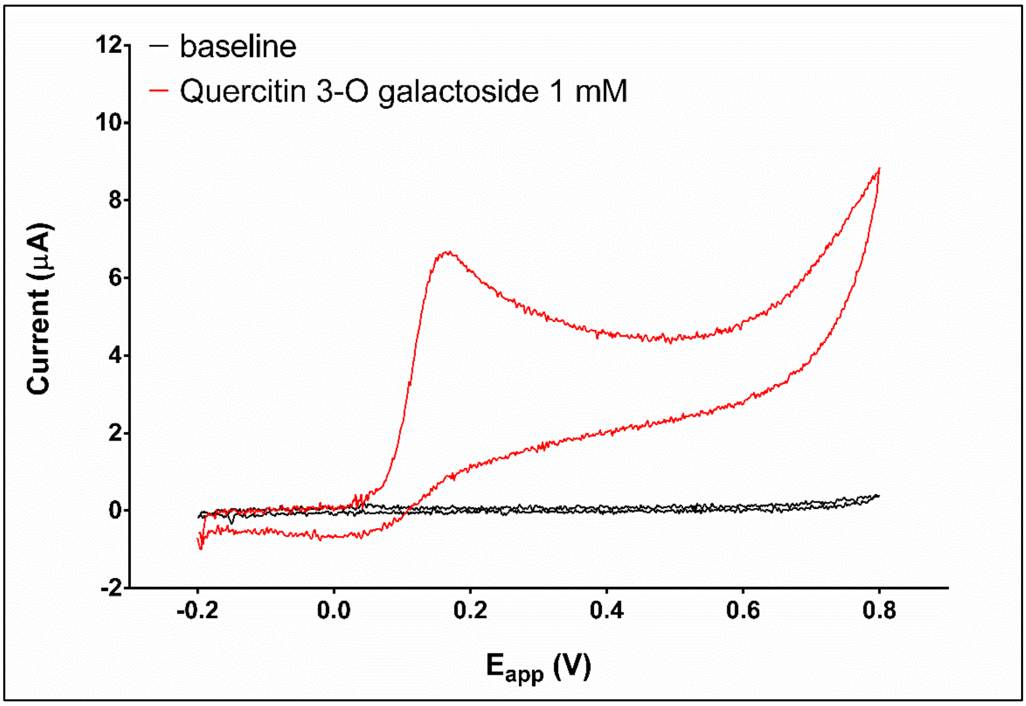

Figure S8. Cyclic voltammograms with a scanned potential range (E_{app}) comprised between -1 V and +1 V, in the absence (black line) and in the presence of 1 mM quercetin 3-*O* galactoside (red line)

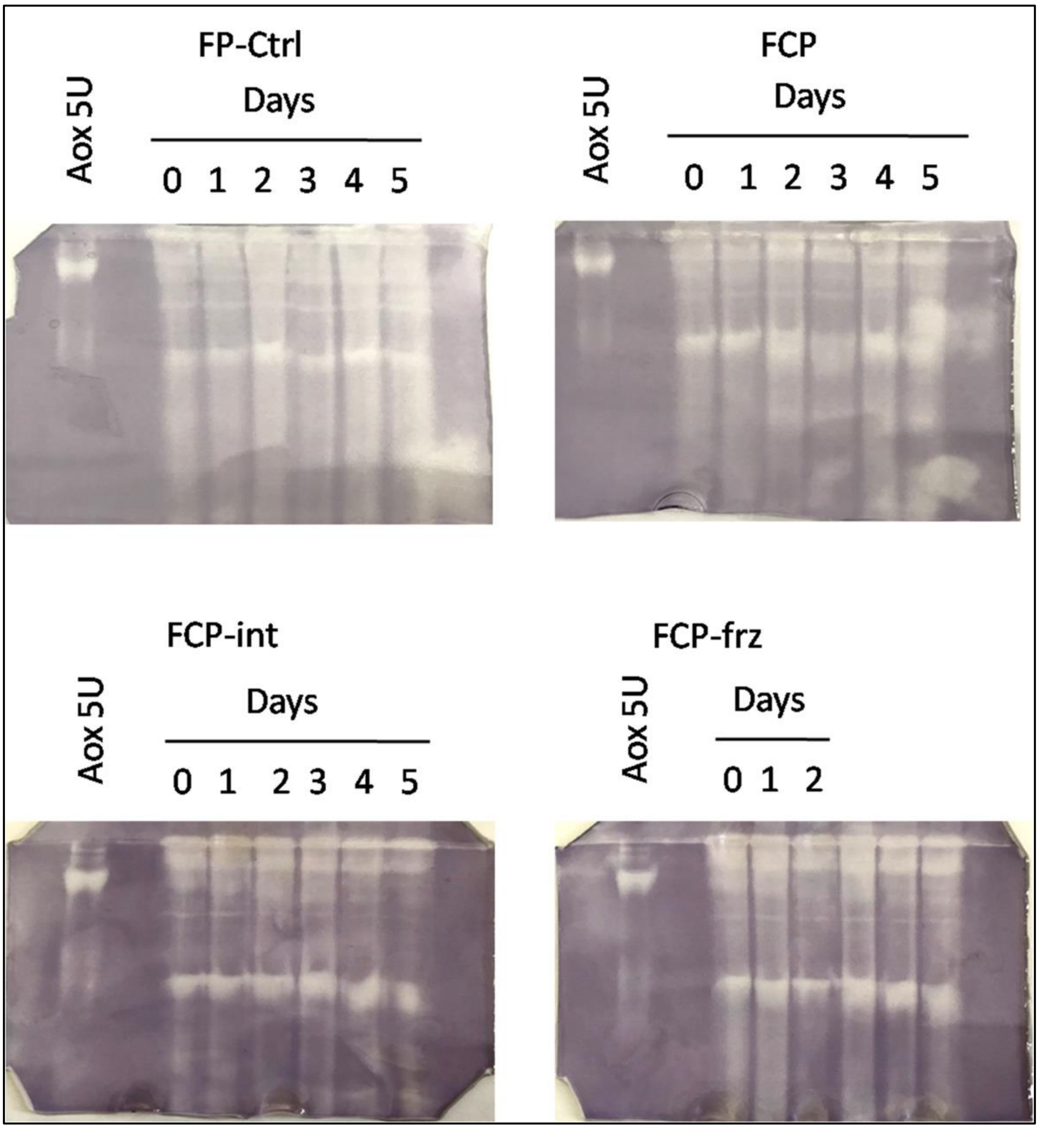

Figure S9. Uncropped full-length native gel pictures relative to changes in AOx activity in figure 5 in the main manuscript.

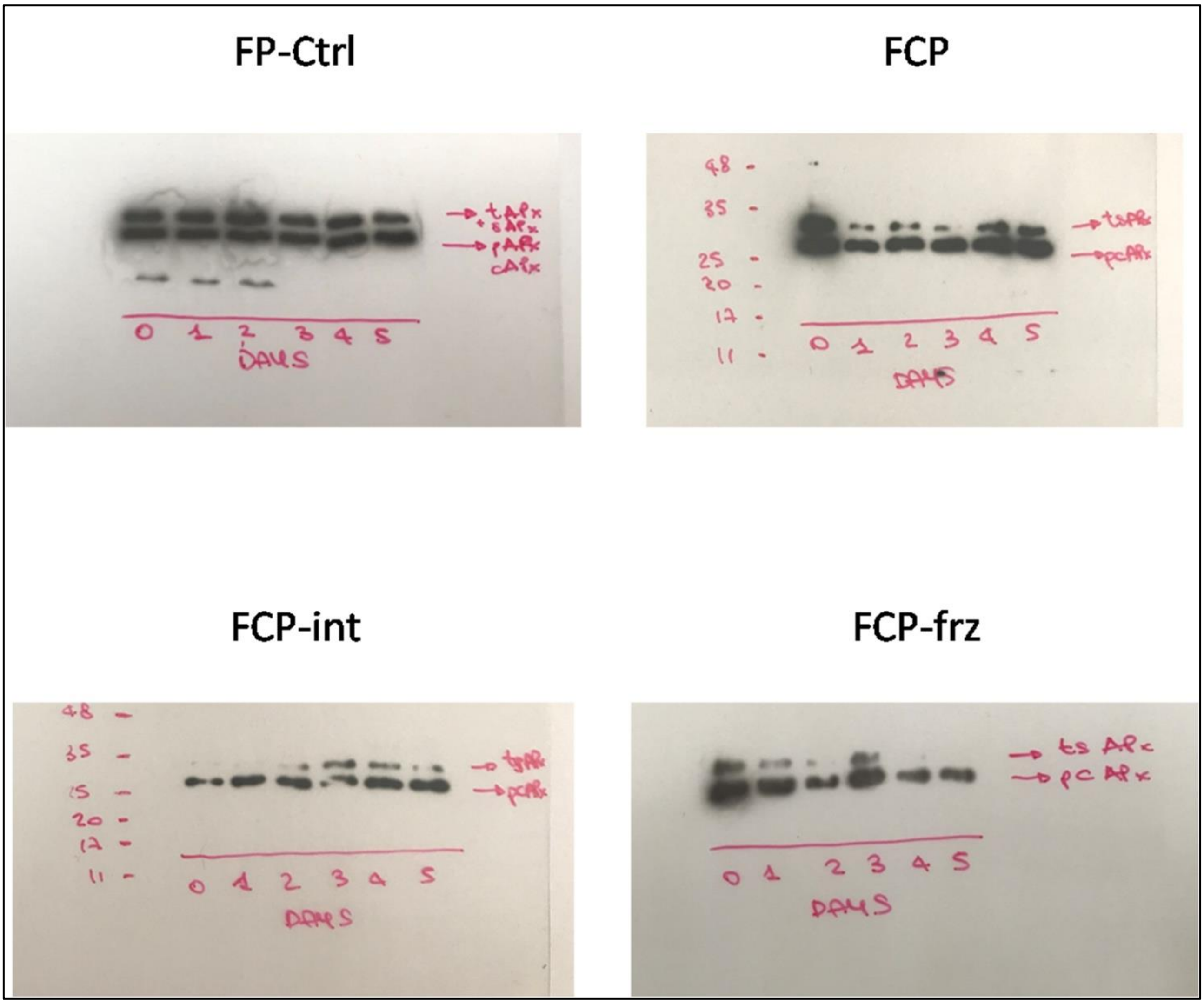

Figure S10. Uncropped full-length western blot gel pictures relative to changes in APx protein expression in figure 6 in the main manuscript.

Table S1. Changes in AOx activity in fresh and fresh-cut parsley under different storage conditions, expressed as AOx enzyme unit (1 unit will oxidize 1.0 μmole of L-ascorbate to dehydroascorbate per min at pH 5.6 at 25 °C). Values are means ± standard deviation, n = 3. Means in columns followed by unlike letters differ significantly by Fisher's least significant difference (LSD) test, $p \leq 0.05$. Means in rows followed by (unlike letters) differ significantly by Fisher's least significant difference (LSD) test, $p \leq 0.05$.

Ascorbate Oxidase Enzyme Units

	Day 0	Day 1	Day 2	Day 3	Day 4	Day 5
**FP-Ctrl**	2.796 ± 0.020 a (c)	2.463 ± 0.080 a (d)	2.796 ± 0.062 a (c)	3.290 ± 0.012 a (b)	3.719 ± 0.035 a (a)	3.927 ± 0.025 a (a)
**FCP**	1.566 ± 0.016 b (c)	1.160 ± 0.050 b (d)	2.058 ± 0.042 b (b)	2.056 ± 0.065 b (b)	2.710 ± 0.023 b (a)	2.074 ± 0.034 b (b)
**FCP-int**	0.706 ± 0.032 c (a)	0.641 ± 0.030 c (a)	0.578 ± 0.032 c (a)	0.524 ± 0.032 c (a)	0.372 ± 0.063 c (b)	0.363 ± 0.031 c (b)
**FCP-frz**	0.841 ± 0.015 c (a)	0.635 ± 0.040 c (b)	0.096 ± 0.012 d (c)			

Table S2. Changes in APx expression in fresh and fresh-cut parsley under different storage conditions, according to the optical density of APx protein separated with western blot technique. The expression of thylakoid and stromal (tsAPx), peroxisome and cytosolic (pcAPx) isoforms, as well as the total APx, is shown in the table. Values are means ± standard deviation, n = 3. Means in columns followed by unlike letters differ significantly by Fisher's least significant difference (LSD) test, $p \leq 0.05$. Means in rows followed by (unlike letters) differ significantly by Fisher's least significant difference (LSD) test, $p \leq 0.05$.

tsAPx

	Day 0	Day 1	Day 2	Day 3	Day 4	Day 5
**FP-Ctrl**	0.094 ± 0.012 b (a)	0.079 ± 0.008 a (a)	0.088 ± 0.006 a (a)	0.076 ± 0.006 a (a)	0.080 ± 0.003 a (a)	0.073 ± 0.003 a (a)
**FCP**	0.150 ± 0.003 a (a)	0.024 ± 0.007 c (c)	0.035 ± 0.002 b (c)	0.025 ± 0.010 c (c)	0.072 ± 0.005 a (b)	0.078 ± 0.007 a (b)
**FCP-int**	0.017 ± 0.004 c (c)	0.019 ± 0.002 c (c)	0.030 ± 0.003 b (b)	0.044 ± 0.003 b (a)	0.039 ± 0.005 b (a)	0.029 ± 0.003 b (b)
**FCP-frz**	0.086 ± 0.009 b (a)	0.046 ± 0.007 b (b)	0.037 ± 0.009 b (b)			

pcAPx

	Day 0	Day 1	Day 2	Day 3	Day 4	Day 5
**FP-Ctrl**	0.084 ± 0.006 b (a)	0.068 ± 0.007 c (a)	0.088 ± 0.003 b (a)	0.086 ± 0.005 a (a)	0.073 ± 0.007 b (a)	0.092 ± 0.004 b (a)
**FCP**	0.170 ± 0.004 a (a)	0.090 ± 0.004 b (c)	0.121 ± 0.009 a (b)	0.076 ± 0.007 a (c)	0.163 ± 0.006 a (a)	0.162 ± 0.002 a (a)
**FCP-int**	0.052 ± 0.005 c (a)	0.057 ± 0.009 c (a)	0.069 ± 0.005 bc (a)	0.053 ± 0.004 b (a)	0.064 ± 0.007 b (a)	0.068 ± 0.003 c (a)
**FCP-frz**	0.190 ± 0.007 a (a)	0.126 ± 0.001 a (b)	0.064 ± 0.003 c (c)			

total APx

	Day 0	Day 1	Day 2	Day 3	Day 4	Day 5
**FP-Ctrl**	0.178 ± 0.006 c (a)	0.146 ± 0.004 a (a)	0.177 ± 0.003 a (a)	0.162 ± 0.001 a (a)	0.153 ± 0.009 b (a)	0.165 ± 0.007 b (a)
**FCP**	0.320 ± 0.001 a (a)	0.114 ± 0.006 b (d)	0.156 ± 0.004 b (c)	0.101 ± 0.002 b (e)	0.235 ± 0.004 a (b)	0.240 ± 0.009 a (b)
**FCP-int**	0.069 ± 0.002 d (b)	0.076 ± 0.004 c (b)	0.099 ± 0.003 c (a)	0.098 ± 0.007 b (a)	0.103 ± 0.001 c (a)	0.097 ± 0.005 c (a)
**FCP-frz**	0.276 ± 0.004 b (a)	0.172 ± 0.001 a (b)	0.101 ± 0.003 c (c)			

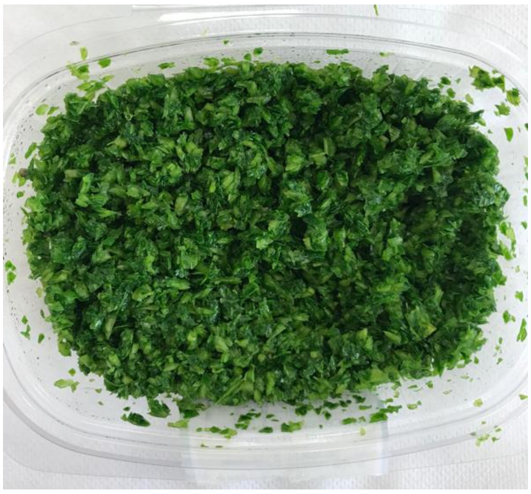 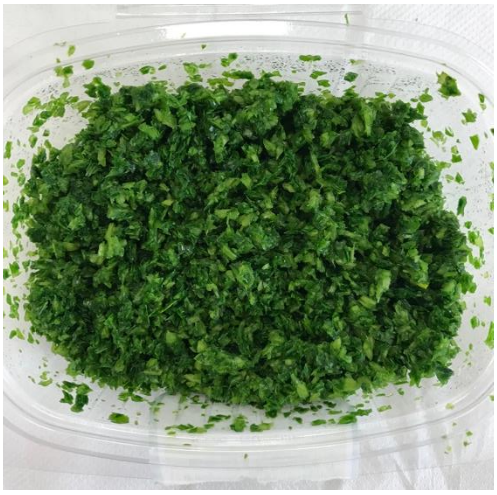

Figure S11. Fresh-cut parsley packaged in 80 g see-through resealable polypropylene trays for foodstuffs. Image taken at day 1, 24 hours after packaging. The image on the left represents fresh-cut parsley correctly stored. The one on the right was subjected to two hours freezing at -2 °C after processing. No visual differences can be observed, and no smell differences were perceived.
